# Special Issue: ‘Advances in Space Biology’

**DOI:** 10.3390/life14080931

**Published:** 2024-07-25

**Authors:** Claudia Pacelli, Francesca Ferranti, Marta Del Bianco

**Affiliations:** 1 Italian Space Agency, Via del Politecnico s.n.c., 00133 Rome, Italy; 2 Centre for Space Life Sciences, Viale Regina Elena, 299, 00161 Rome, Italy

As we enter a new era of space exploration, space biology is at the forefront of both robotic and human space programs [1,2]. Unmanned missions to Venus and the icy moons of Jupiter and Saturn will be the next goals in space travel for the investigation of the origins of life and search for life beyond Earth. On the other hand, international space agencies are taking steps to push the boundaries of human exploration beyond Low Earth Orbit (LEO), with the aim of establishing a permanent human presence on the Moon and bringing humans to the surface of Mars. However, with these new exploration horizons come new challenges, not only from technological and engineering points of view, but also, most importantly, from a biological and biomedical prospective. The environment in space is characterized by unfavorable conditions, like altered gravity, high doses of radiation, and isolated and confined environments [3,4,5,6,7,8]. These conditions have been experienced with increasing duration by humans and many biological systems since the beginning of the space race, starting from the first flights to the International Space Station (ISS) in LEO. To guarantee a safe and successful human space exploration program, space agencies aim to draft a comprehensive risk model and design effective countermeasures. With this in mind, space biology research aims to understand how life can survive in space, through experiments performed in either simulated or true space conditions [4]. To achieve this goal, new methodologies are constantly being developed to tackle the constraints of conducting experiments in space [8,9].

Since the beginning of the human space exploration era, it has been evident that mechanical unloading due to microgravity conditions is detrimental to the musculo-skeletal system [10]. Despite the numerous investigations performed on this topic so far [10,11], the molecular mechanisms related to muscle atrophy and bone loss are not yet fully understood. Cariati et al. [12] studied the influence of simulated microgravity on the mineralization process using the SAOS-2 cell line, investigating changes in cellular mineralization capability and in the expression of the positive regulator of bone mineralization PTX3. The authors showed that the effects of simulated microgravity depend on the presence or absence of osteogenic factors. In non-osteogenic conditions, simulated microgravity increased cells’ viability and improved their mineralization competence. When cells were not treated with an osteogenic cocktail, simulated microgravity negatively affected the mineralization process, as evidenced by the reduced presence of calcification-like structures, calcium deposits, and PTX3 expression. The acute response to microgravity acts at various levels: molecular, cellular, tissue, and physiological. In particular, the mechanical solicitation perceived within the cell (cytoskeleton, nucleoskeleton), at the membrane (focal adhesion, cell junctions), and outside the cell (extracellular matrix) may contribute to the response to altered gravity at the cellular level. Andreeva et al. [13] reviewed the changes in the extracellular matrix properties of stromal and skeleton tissues induced by real and simulated microgravity, highlighting their role in the loss of bone mass and the need for more focused studies on this topic. Moreover, it was suggested that microgravity negatively regulates the formation of focal adhesion with the extracellular matrix in human neural stem progenitor cells (hNSPCs), causing further transcriptional and morphological changes, which resemble the effects of low-density culture [14]. Taken together, these works again confirm the importance of extracellular matrix components as the main gravity-sensitive structures outside the cell.

There are strong parallels between the molecular mechanisms involved in age-related issues and the effects of space on human health [15]. For example, microgravity-induced muscle atrophy shares molecular mechanisms with sarcopenia and frailty in elderly people [16]. The altered structural and functional integrity of the skeletal muscle observed in older people and in astronauts can be ascribed to increased inflammation, reactive oxygen species (ROS)/reactive nitrogen species (RNS), and altered myokine expression. Countermeasures based on nutritional supplementation, pharmacological intervention, and physical exercise can help to reduce oxidative damage and inflammation and stabilize myokine expression [17]. As for physical performance, the confinement, isolation, and overall psychophysical stress experienced on spacecraft/space stations can affect memory and performance in astronauts in a similar way to aging on Earth. Mammarella et al. [18] attempt to outline the genetic profile related to the positivity effect on memory. This contribution identifies a series of genes that, alone or in combination, may modulate the maintenance of good memory in older adults, pointing out individuals who are better able to adapt to stressful situations. It should be noted that microgravity can affect the behavior of an individual by inducing a stress response at the cellular level. Rubinstein et al. [19] show that simulated inactivity triggers a mitochondrial response mediated by ROS signaling in the hippocampus, which is associated with a decrease in exploratory behavior. In this context, the effects of space radiation, which also elicit a ROS-mediated stress response, on the human central nervous system should also be taken into account [20]. Scatà et al. [21] showed that social isolation activated the autonomic nervous system, enhancing the cardiovascular response and activating the transcription of pro-inflammatory genes, which led to inflammation. This response is adaptive in the short term, but it also requires a rapid return to the initial state of equilibrium in order to prevent the detrimental effects of prolonged activation, such as cardiovascular impairment and immune imbalance. On Earth, it has become evident that social isolation, especially among the elderly, might be deleterious for mental health and is related to higher mortality rates. In this context, the lengthening of space missions and Mars exploration, the threat of future pandemics, and the aging of the population all share common challenges that can be highlighted by these space studies. These studies enable us to structure effective countermeasures that could have a great impact on Earth, improving quality of life, especially for elderly people.

In recent years, it has become evident that microgravity can cause changes in human and environmental microbiomes [22,23,24,25]. The skin is an important organ that acts as a barrier to pathogens and helps maintain physiological homeostasis during environmental changes. Alterations in the skin microbiota could result in skin diseases, which could compromise this important skin function. Indeed, astronauts have reported skin irritation and infections, among the other recurring health issues, during space flight [26,27]. Tozzo et al. [28] focus on the less-studied skin microbiome, showing that skin microbiota changes in space appear to be temporary. Interestingly, in a closed habitat, an individual’s skin microbiome tends to reach a balance with the microbiomes of other individuals and that of the environment.

While astrobiology can help us understand the effects of prolonged stays in Space on living organisms, its main focus is on the origin of life on Earth and the search for life beyond Earth [29,30]. This search relies on the remote sensing of biosignatures during unmanned missions. These biosignatures are indicators of past or present life, and are based on the molecular peculiarity of life on Earth and on simulation studies of terrestrial biogenesis. To achieve a plausible computational model, it is crucial to understand the sequence of physico-chemical processes that led to the emergence of life on primitive Earth, which is the only available example of life we know of. In this context, Vladilo [31] investigated the role ^40^K in the origin of terrestrial life during the Archean. The author concludes that the particles emitted by ^40^K decay might be caused of excess L-type amino acids on primitive Earth when life began. Indeed, proteins are good biosignature candidates due to their role in multiple biological functions. However, the remote detection of all biosignatures, including proteins, is challenging. In the search for life on the surface of Mars, novel and simple biochemical methods for detecting proteins and DNA in Martian soils have been suggested [32,33,34]. In the future, these promising technologies could be validated in flight using cost-effective CubeSats [35].

To conclude, the review and original articles presented in this Special Issue “Advances in Space Biology” have significantly contributed to deepening our knowledge of space biology.
